# Medical Relief under the New Poor-Law

**Published:** 1838-01

**Authors:** 


### MEDICAL RELIEF UNDER THE NEW POOR-LAW.

This subject engrosses so much of the attention of the profession at present, and we have received so many communications respecting it, that we think it necessary to state that it is our intention to consider it in detail in an early Number. One of the subjects to which the attention of Parliament will be directed during the present session will be the errors that have crept, more or less, into every part of the Poor-law Amendment Act, and especially into that part of it relating to the medical relief of paupers. Deeming this Act one of the *most* important that has ever been passed by the governing body of any nation, although its large views cannot be accomplished except in connexion with other means of raising and improving the character and condition of the labouring classes, we have refrained from any hasty condemnation of its defects, and have deeply regretted to see its intentions and probable effects so mistaken or so misstated, not only by many public men for party purposes, but, as regards our own profession, by many of its members, and many who profess themselves the best friends of medical men. It is evident to us, as to all, that useful modifications are required, and may easily be made, in those portions of the Bill which relate to medical attendance on paupers; but these modifications have often been urged without temper, and sometimes without consideration. When the whole subject comes regularly before us, we shall examine it freely and impartially, with a sincere regard for the welfare of the profession, but with small respect for their prejudices, and with no dread of mere clamour. In the mean time, we may say that it appears to us that the establishment of fixed salaries in place of the system of *tenders*, the subdivision of districts, and the allotment of them with a more constant regard to the residence of the medical man, are much required. That the remuneration ought to be just and regular, no one will be disposed to deny. The interests of medical men, (than whom none gain a livelihood with more care, labour, and anxiety,) demand these changes; and they are no less demanded by a real humanity, which excludes the spurious humanity from which pauperism has derived so much direct and indirect support.

Although, in the foregoing observations, we have spoken of the defects as having crept into *the law*, the truth is, they are almost exclusively chargeable on the manner in which this has been administered by the boards of guardians. It is also too true that not a little of the harshness of the details of which medical men now complain, and justly complain, have originated in their own jealousies and want of mutual confidence. When this law was first brought into operation, if all the members of the profession in the different districts had met, and consulted cordially and candidly together,—had agreed to sacrifice their own little individual advantages for the common good of the profession,—and, in their intercourse with the Boards, had shewn that they consulted only the *general interests* of the medical body and the *welfare of the poor*, we are persuaded that matters would have worn a very different aspect from what they do at present. It must never be forgotten that this great law was devised, not for the advantage of the medical profession, but for the improvement of the condition of the poor; and if, in the attainment of this paramount advantage, some of the more favoured classes of society suffer temporary loss or inconvenience, surely there is some compensation in the philanthropic and patriotic feelings which attend the sacrifice. It would, indeed, be most strange if the members of the medical profession,—a profession which has always been honorably distinguished for its large and active benevolence,—should hesitate, on such an occasion as the present, to participate, in some slight degree, in those inconveniences and privations which at present bear so hardly on their poorer brethren; but which, it is confidently believed, will eventually be productive of incalculable benefit to their order.

